# Clinical characteristics of perimenopausal migraine and its correlation to hormonal levels

**DOI:** 10.1186/s10194-026-02418-8

**Published:** 2026-06-16

**Authors:** Lobna Talaat El-Ghoneimy, Nervana M. Elfayoumy, Hanan Helmy, Menatallah Mohamed Saleh Elaguizy, Anter Mothenna Abdullah Nasser, Hend Abdelghany

**Affiliations:** Department of Neurology, Cairo University, Cairo, 11562 Egypt

**Keywords:** Perimenopausal migraine, Estradiol, FSH, Migraine headache, Vasomotor symptoms

## Abstract

**Background:**

Studies about perimenopausal migraine are limited. In this study, we aimed to explore the characteristics of perimenopausal migraine and to compare perimenopausal women with and without migraine in terms of the levels of Estradiol and Follicle-Stimulating Hormone (FSH). We also aimed to correlate migraine severity scores with Estradiol and FSH levels.

**Methods:**

A case-control study of 66 subjects divided as Group 1 (*n* = 22): migraine onset during perimenopause, Group 2 (*n* = 22) migraine onset before perimenopause, and Group 3 (*n* = 22) age-matched healthy controls. Perimenopausal women with any type of migraine (age 40–55 years) were eligible. We excluded secondary and other primary headaches, hormonal therapy and chronic illness. Subjects underwent a detailed history taking of headache and vasomotor symptoms, MIDAS and VAS scores. Measurement of Estradiol (E2) and FSH levels was done for all participants. Comparisons were done between group 1 and group 2 by migraine characteristics, vasomotor symptoms and Estradiol and FSH levels. Group 3 (control) was compared with both migraine groups 1 and 2 by vasomotor symptoms and Estradiol and FSH levels. Statistical analysis was performed by using SPSS 27th edition Statistical tests used include mean, median, count, Mann-Whitney U, Kruskal-Wallis test, Chi-squared test and the Spearman correlation test (The suitable test was chosen based on the type of variables and data, and the number of comparison groups).

**Results:**

Migraine with onset during perimenopause was characterized by throbbing pain, alternating laterality, orbito-fronto-temporal site, moderate to severe intensity, high frequency, with associated phonophobia and photophobia, while autonomic symptoms were rare. There was analgesic overuse and an unsatisfactory response. Group 2 significantly had more postdrome symptoms, less analgesic response and more menstrually-triggered migraine than Group 1 (P < 0.05). Both perimenopausal migraine groups (Group 1 and 2) had significantly more perimenopausal symptoms, including fatigue, sleep disturbance, mood symptoms and anxiety (P < 0.05) compared to the control (Group 3). Hot flashes, night sweating and vaginal dryness were more common in migraineurs without statistical significance. Migraineurs had significantly lower Estradiol levels and a higher proportion of subjects with Estradiol < 50 pg./ml than controls (P < 0.05). There was a significant negative correlation between Estradiol and scores, including MIDAS (Rho=-0.406, *P* = 0.007) and VAS (Rho= -0.331, *P* = 0.030). No difference in the FSH levels among the Groups.

**Conclusion:**

Perimenopause is associated with high-frequency, moderate to severe migraine headache. Lower Estradiol levels were correlated with high migraine severity scores (VAS and MIDAS). The frequency of perimenopausal symptoms is higher, whereas the Estradiol level is lower in migrainous compared to nonmigrainous women.

## Introduction

Migraine has great adverse effects on the quality of life of its sufferer [1]. There is a discrepancy in the prevalence of this disorder among different countries, which may be attributed to racial, genetic, lifestyle and environmental factors. It was estimated that migraine affect about 14–15% of the population worldwide [2]. There is a scarcity of data regarding the prevalence of migraine in Egypt. However, the one-year migraine prevalence was reported to be 17.3% by a community-based observational study conducted in Fayoum governorate, Egypt [3]. Another study conducted in Al Quseir city, Egypt, had shown that the one-year migraine prevalence was 2800/100,000 for those aged more than 8 years [4]. In fact, migraine is more common in females, with a cumulative lifetime frequency of 43% in females versus 18% in males, with an estimated female: male ratio of 3:1, according to the American Migraine Prevalence and Prevention study. The relatively higher female predominance of migraine can be due to the effect of sex hormones [5]. The fluctuation of female sex hormones may have a relevant effect on migraine headache, as evidenced by changes in migraine frequency, pain intensity and drug response around the menstrual period, menopausal phase, during pregnancy and when using oral contraceptive pills or hormonal replacement therapy [6]. Migraine is a sequel of the trigeminovascular activation and the release of many chemicals like “calcitonin gene-related peptide (CGRP)” and “pituitary adenylate cyclase-activating polypeptide (PACAP)” [7].

The perimenopausal period is associated with significant instability in the estrogen levels, predisposing to a higher incidence of migraine headaches. Additionally, the typical anovulation seen during the menopausal transition period may be a trigger for migraine. On the contrary, the postmenopausal period, which is characterized by hormonal stability due to the reduced release of these hormones, is associated with an improvement in migraine attacks [8]. Unfortunately, the effects of various treatment regimens on perimenopausal migraine have also been studied, with inconclusive results. Too far, there are no specific preventive strategies for menopausal migraine in the current migraine guidelines [5]. There are limited studies, heterogeneity of data, a great debate and conflicts in the literature regarding migraine profile during the perimenopausal and the postmenopausal age group. The issue of migraine headache and women’s life cycle transitions (particularly the perimenopausal age) is a great focus of recent research. Furthermore, migraine characteristics can vary between different countries due to variation in race, lifestyle, life stressors that women face, and environmental factors [9]. For these reasons, it is crucial to conduct specific studies of migraine in a specific population. In this study, we aimed to correlate the severity of migraine with the plasma levels of Estradiol and Follicle-Stimulating Hormone (FSH). We also aimed to explore the characteristics of migraine in the perimenopausal period and to compare migrainous and non-migrainous perimenopausal women with regard to the hormonal levels of Estradiol and FSH.

## Methods

This is a case-control study that was conducted between February 2024 and May 2025 at Cairo University Teaching Hospital, Cairo, Egypt. We aimed to correlate between the severity of migraine and the plasma levels of Estradiol and follicle-stimulating hormone (FSH) as the primary outcome of this study. The secondary outcome is to explore the characteristics of migraine in the perimenopausal period and to compare migrainous and non-migrainous perimenopausal women with regard to the hormonal levels of Estradiol and FSH.

### Study subjects and sampling

This is a case-control study of 66 subjects, recruited from the Outpatient Headache Clinic of the Neurology Department, Kasr Alainy Hospital, Cairo University, Egypt. The subjects were divided into three groups: Group I, which included perimenopausal patients who were proven to have migraine headache with an onset during their perimenopausal period, Group II, which included perimenopausal patients who were proven to have migraine headache with an onset during their childbearing age. Group II will be used as an active comparator group with Group I in order to further characterize the perimenopausal-onset migraine. This comparison will enable us to assess if migraine severity and hormonal profiles in the perimenopausal onset migraine subjects differ from those observed in women with long-lasting migraine who are currently going into the perimenopausal period. The third group is Group III (Control group), which included apparently healthy perimenopausal participants matched in age to the patient groups. Group III will be compared with Groups I and II regarding the vasomotor symptoms and hormonal levels (Estradiol and FSH).

The minimum required sample size was calculated using SAS Institute Inc., 2013. SAS/ACCESS^®^ 9.4 Interface to ADABAS: Reference. Cary, NC: SAS Institute Inc. Using a power of 80%, 95% confidence interval, expected correlation between Estradiol level and VAS score of headaches (*r* = 0.583) as previously reported in literature [10] and 0.05 level of significance. We found that the minimum required sample size for this study is 20 patients per group. With an allocation ratio of 1:1:1, patients will be divided into three groups: Group 1: 20 patients who developed migraine within the childbearing age. Group 2: 20 patients who developed migraine within the perimenopausal state. Group 3: 20 normal healthy perimenopausal controls.

### Inclusion and exclusion criteria

The inclusion criteria of Group I include females, with an age range from 40 to 55 years, who were diagnosed as perimenopausal according to the NICE guidelines, 2017 and the Stages of Reproductive Aging Workshop + 10 (STRAW + 10 criteria). [Perimenopause is defined as the transition period between childbearing age and menopause which is characterized by menstrual irregularities (of more than 7 days change per cycle) or unexplained secondary amenorrhea of more than 60 days and less than 12 months duration, plus vasomotor symptoms (If the age is ≥ 45 years there is no need for FSH testing, while if the age is ≤ 45 years, raised FSH level is recommended to diagnose perimenopause)] [11, 12]. We included new patients who attended the clinic for the first time, who had either episodic or chronic migraine, with/without aura, diagnosed according to criteria of the International Classification of Headache Disorders, 3rd edition (2018) [13], with migraine onset during the perimenopausal period. The inclusion criteria of Group II include females, with an age range from 40 to 55 years, who were diagnosed as perimenopausal according to the NICE guidelines, 2017 and STRAW + 10 criteria [11, 12]. New patients who attended the clinic for the first time, who had either episodic or chronic migraine, with/without aura, diagnosed according to the criteria of the International Classification of Headache Disorders, 3rd edition (2018) [13], with migraine onset before the perimenopausal period. The inclusion criteria of Group III include females, with an age range from 40 to 55 years, who were diagnosed as perimenopausal according to the NICE guidelines, 2017 and STRAW + 10 criteria [11, 12] and who are healthy, have no migraine headache and are age-matched to migraine groups I and II. All participants in the three groups were still in the perimenopausal phase during assessment and recruitment.

The exclusion criteria of Group I and II include: other primary headaches, secondary etiologies of headache as listed in the ICHD-3, 2018 [13], red flags of headache [14], surgical menopause, organic gynecological disorders (Polycystic ovary syndrome, ovarian tumors), use of migraine preventive therapy or hormonal replacement therapy (HRT) and chronic medical illness (hypertension, diabetes mellitus, liver disease, kidney disease, endocrine disease or malignancy). Participants with migraine onset during the perimenopausal period were excluded from Group II, whereas those who had migraine onset before perimenopause were excluded from Group I.

We excluded from Group III (control) those who didn’t fulfill the criteria for diagnosis of perimenopause, those who have any type of headache, surgical menopause, organic gynecological disorders (Polycystic ovary syndrome, ovarian tumors), use of hormonal replacement therapy (HRT) and chronic medical illness (hypertension, diabetes mellitus, liver disease, kidney disease, endocrine disease or malignancy).

### Data collection tools and procedure

We conducted face-to-face interviews to include subjects according to inclusion and exclusion criteria, then a detailed history taking was taken from the patients, including demographic data, migraine characteristics (onset, course, duration, frequency, site, laterality, pain nature, triggers and relieving factors, associated symptoms, severity, analgesics used, premonitory and postdrome symptoms, migraine change during climacteric). We had assessed the menstrual status for the presence of menstruation, menstrual irregularities and time from the last menstrual period (must be less than 12 months to exclude menopause). We also assessed the perimenopausal symptoms (hot flashes, night sweating, vaginal dryness, depressive mood, anxiety, fatigue and insomnia).

Clinical Scores that were used include the Validated Arabic version of Migraine Disability Assessment Questionnaire (MIDAS) to assess the functional disability [15]. The intensity of pain was assessed by the Visual Analogue Scale (VAS) [16].

Measurement of Estradiol (E2) and FSH levels was done for all participants. Blood samples were withdrawn irrespective of the menstrual cycle phase (due to irregular menses during perimenopause). Estradiol and FSH were measured in serum through the electrochemiluminescence immunoassay (ECLIA) technique on Cobas e601 Immunoassay Analyzer.

### Statistical analysis

Statistical analysis was performed by using SPSS 27th edition. The qualitative variables were presented in count and percentage, while the quantitative variables were presented in mean, standard deviation, median, minimum and maximum. To compare quantitative variables between two groups, the Mann-Whitney U test was used, while comparing them between more than two groups, the Kruskal-Wallis test was used. Chi-squared test was used to compare categorical variables between more than two groups. The Bonferroni correction formula was used for the correction of the P value in multiple testing. The linear correlation between two quantitative variables was assessed by the Spearman correlation test. Any P-value < 0.05 was considered significant.

## Results

In this case control study, a total of 66 participants were enrolled, divided into three Groups: Group I (*n* = 22, perimenopausal migraine with an onset during their perimenopausal period), Group II (*n* = 22, perimenopausal migraine with an onset during their child-bearing age and Group III (*n* = 22, Control Group).

### Comparison of demographics and characteristics of migraine between Group 1 and Group 2

The mean age of Group 1 is 46.6 ± 2.5 year, and that of Group 2 is 47.1 ± 2 year. BMI was slightly higher in Group 1 (33.3 ± 7.3 kg/m^2^ versus 30.5 ± 5 kg/m^2^) but not statistically significant. There is no statistically significant difference between the two groups regarding their age, occupation, family history and smoking, indicating matched lifestyle profiles. Although the monthly migraine days were higher in Group 2, this difference didn’t reach statistical significance. Notably, the other migraine characteristics, such as attack duration and headache-free intervals, were statistically comparable. The location, side, and character of headaches were also similar, with pulsating pain being the most frequent in both Groups. Common associated features like osmophobia, photophobia, phonophobia, nausea/vomiting and allodynia were prevalent in both Groups with no significant differences, suggesting that while onset time differs, core migraine features remain similar **(**Table 1**).**

**Table 1:** Comparison of demographics and disease characteristics between perimenopausal migraine groups 1 and 2

		Group 1^a^ (*n* = 22)		Group 2^a^ (*n* = 22)		*P* value*
		Mean ± SD	Median (Min, Max)	Mean ± SD	Median (Min, Max)	
Age (years)		46.6 ± 2.5	-	47.1 ± 2	-	0.073
BMI (Kg/m2)		33.3 ± 7.3	-	30.5 ± 5	-	0.218
Duration of headache (years)		-	3 (1, 10)	-	12.5 (2, 32)	< 0.001
Duration of attack (hours)		-	8 (1.5, 48)	-	6 (3, 24)	0.635
Monthly migraine days (MMD)		-	12 (2, 24)	-	12 (3, 30)	0.662
Headache free days		-	10 (1, 90)	-	17 (0, 180)	0.680
		Count	%	Count	%	
Laterality	unilateral	6	27.3%	4	18.2%	0.753
	Bilateral	5	22.7%	5	22.7%	
	Alternating	11	50.0%	13	59.1%	
Site	Global	5	22.7%	2	9.1%	0.446
	Orbito-frontal	6	27.3%	8	36.4%	
	Orbito-fronto-temporal	11	50.0%	12	54.5%	
Character	Pulsating	20	90.9%	21	95.5%	0.220
	Pressing	2	9.1%	0	0.0%	
	Aching	0	0.0%	1	4.5%	
Osmophobia	Yes	4	18.2%	4	18.2%	1.000
	No	18	81.8%	18	81.8%	
photophobia	Yes	20	90.9%	21	95.5%	0.550
	No	2	9.1%	1	4.5%	
phonophobia	Yes	21	95.5%	20	90.9%	0.550
	No	1	4.5%	2	9.1%	
Nausea /Vomiting	Yes	14	63.6%	15	68.2%	0.750
	No	8	36.4%	7	31.8%	
Autonomic symptoms	Yes	2	9.1%	3	13.6%	0.635
	No	20	90.9%	19	86.4%	
Allodynia	Yes	12	54.5%	13	59.1%	0.761
	No	10	45.5%	9	40.9%	
Family history	Positive	8	36.4%	8	36.4%	1.000
	Negative	14	63.6%	14	63.6%	
Smoking	Yes	1	4.5%	2	9.1%	0.550
	No	21	95.5%	20	90.9%	
Education	Uneducated	5	22.7%	11	50%	0.060
	Educated	11	77.3%	11	50%	
Occupation	Unemployed	4	18.2%	4	18.2%	1.000
	Employed	18	81.8%	18	81.8%	

BMI: body mass index, Kg: kilogram, MMD: Monthly Migraine days, M^2^: square meter, SD: standard deviation. * p value < 0.05 is considered significant

^a^ Group 1 includes perimenopausal patients who were proved to have migraine headache with an onset during their perimenopausal period while Group 2 includes perimenopausal patients who were proved to have migraine headache with an onset during their child-bearing age

A higher percentage of Group 2 patients reported severe migraines (77.3%) compared to Group 2 (59.1%), though the difference wasn’t statistically significant (*p* = 0.195). Aura was more common in Group 2 (17.2%) than Group 1 (4.5%), approaching significance (*p* = 0.079), with visual aura being most frequent. Status migrainosus was not significantly different between Groups. The distribution of migraine types showed that episodic migraine is slightly more common in Group 1 (68.2%) compared to Group 2 (59.1%), while chronic migraines are higher in Group 2 (40.9%) compared to Group 1 (31.8%), with no statistically significant difference (P value = 0.535). Notably, postdrome symptoms (e.g., fatigue, mood changes following migraine resolution) were more common in Group 2 (18.2%) than in Group 1 (50%) with a statistically significant difference (P value = 0.026) **(**Table 2**).**

**Table 2:** Comparison of types, premonitory and postdrome symptoms of migraine between Group 1 and Group 2

		Group 1^a^ (*n* = 22)		Group 2^a^ (*n* = 22)		P value
		Count	%	Count	%	
Migraine with Aura	Yes	1	4.5%	4	17.2%	0.079
	No	21	95.5%	18	81.8%	
Type of aura	No	21	95.5%	18	81.8%	0.156
	vestibular	0	0.0%	1	4.5%	
	visual	1	4.5%	3	13.6%	
Status migrainosus	Yes	3	13.6%	5	22.7%	0.434
	No	19	86.4%	17	77.3%	
Type of migraine	Episodic	15	68.2%	13	59.1%	0.535
	Chronic	7	31.8%	9	40.9%	
Premonitory symptoms	Yes	4	18.2%	11	50.0%	0.434
	No	18	81.8%	11	50.0%	
Postdrome symptoms	Yes	4	18.2%	11	50.0%	0.026*
	No	18	81.8%	11	50.0%	

*P value < 0.05 is considered significant

^a^ Group 1 includes perimenopausal patients who were proved to have migraine headache with an onset during their perimenopausal period while Group 2 includes perimenopausal patients who were proved to have migraine headache with an onset during their child-bearing age

Both groups reported similar percentages for triggers like exertion, fatigue, stress, hot weather, and sleep changes (insomnia). However, Group 2 reported significantly higher frequency of menstrually-triggered migraine attacks than Group 1 (*P* = 0.016). There is no significant difference between both Groups 1 and 2 regarding the relieving factors (Table 3).

**Table 3:** Comparison of precipitating and relieving factors between Group 1 and Group 2

Precipitating factors		Group 1^a^ (*n* = 22)		Group 2^a^(*n* = 22)		*P* value*
		Count	%	Count	%	
Exertion	Yes	18	81.8%	18	81.8%	1.000
	No	4	18.2%	4	18.2%	
Fatigue	Yes	12	54.5%	13	59.1%	0.761
	No	10	45.5%	9	40.9%	
Stress	Yes	20	90.9%	19	86.4%	0.635
	No	2	9.1%	3	13.6%	
Hot weather	Yes	13	59.1%	13	59.1%	1.000
	No	9	40.9%	9	40.9%	
Sleep changes	Yes	10	45.5%	10	45.5%	1.000
	No	12	54.5%	12	54.5%	
Menstrually related migraine	Yes	5	22.7%	13	59.1%	0.016*
	No	17	77.3%	11	40.9%	
fasting	Yes	8	36.4%	12	54.5%	0.226
	No	14	63.6%	10	45.5%	
Relieving factors						
Analgesics	Yes	17	77.3%	16	72.7%	0.729
	No	5	22.7%	6	27.3%	
Dark room	Yes	5	22.7%	8	36.4%	0.322
	No	17	77.3%	14	63.6%	
Sleep	Yes	3	13.6%	2	9.1%	0.635
	No	19	86.4%	20	90.9%	
Bed rest	Yes	4	18.2%	1	4.5%	0.154
	No	18	81.8%	21	95.5%	

*P value < 0.05 is considered significant

^a^ Group 1 includes perimenopausal patients who were proved to have migraine headache with an onset during their perimenopausal period while Group 2 includes perimenopausal patients who were proved to have migraine headache with an onset during their child-bearing age

The median Migraine Disability Assessment Score (MIDAS) in Group 1 was 27, while in Group 2 it was 26.5, and the difference is not statistically significant (*P* = 0.459). VAS scores, which assess migraine pain intensity, were nearly identical between the two Groups: Group 1 recorded a mean score of 6.6 ± 1.3, and Group 2 reported 6.5 ± 1.4. Further categorical analysis of MIDAS scores revealed that both Groups had an equal proportion (31.8%) of participants with moderate disability (11–20), while severe disability (> 20) was slightly more common in Group 2 (59.0%) than Group 1 (54.5%), though this difference was not significant (*P* = 0.765) **(**Table 4**).**

**Table 4:** Comparison of migraine scores between Group 1 and Group 2

		Group 1^a^ (*n* = 22)		Group 2^a^ (*n* = 22)		*P* value*
		Mean ± SD	Median (Min, Max)	Mean ± SD	Median (Min, Max)	
MIDAS (Arabic)		30.4 ± 22.1	27 (0, 77)	35.4 ± 25.7	26.5 (9, 87)	0.459
VAS scale		6.6 ± 1.3	6.0 (4, 9)	6.5 ± 1.4	6.0 (3.8, 9)	0.887
**MIDAS category**		Count	%	count	%	
0–5	Minimal	3	13.6%	0	0%	0.076
6–10	Mild	0	0	2	9.0%	0.154
11–20	Moderate	7	31.8%	7	31.8%	1.000
> 20	Severe	12	54.5%	13	59.0%	0.765
**VAS category**						
< 3	Mild	0	0%	0	0%	1.000
4–6	Moderate	12	54.5%	11	50.0%	0.767
7–10	Severe	10	45.5%	11	50.0%	0.757

*P value < 0.05 is considered significant

^a^ Group 1 includes perimenopausal patients who were proved to have migraine headache with an onset during their perimenopausal period while Group 2 includes perimenopausal patients who were proved to have migraine headache with an onset during their child-bearing age

MIDAS (Arabic): Arabic version of Migraine Disability Assessment Questionnaire, VAS: Visual analogue scale

### Comparison of demographics, perimenopausal symptoms and hormones between migrainous and nonmigrainous participants

Hot flashes were more common in the study Groups (both 81.8%) compared to controls (63.6%), but this difference was also not statistically significant (*p* = 0.267). Vaginal dryness showed a progressive increase from the control Group (22.7%) to Group 1 (36.4%) and Group 2 (50.0%), although this trend did not reach statistical significance (*p* = 0.171). Night sweating was reported by half of the control Group (50.0%) and higher in Group 1 (81.8%) and Group 2 (72.7%), nearing statistical significance (*p* = 0.066). There are statistically significant differences in fatigue, sleep changes (insomnia), depressed mood and anxiety between migraine groups (Group 1 and 2) on one side (reported higher frequency of these symptoms) compared to the control (Group 3) on the other side (p-values ˂0.002). Estradiol levels are notably lower in Group 2 (mean = 23.6 pg./ml) and Group 1 (mean = 24.5pg/ml) compared to Controls (mean = 46.9 pg/ml), with a statistically significant difference (P value = 0.018). Among the hormone thresholds, 84.9% of all participants had Estradiol < 50 pg./ml, more prevalent in Groups 1 and 2 (95.4% and 90.9% respectively) compared to the Control Group (68.2%) with a statistically significant difference (*P* = 0.016). No significant difference in the FSH level among all study groups **(**Table 5**).**

**Table 5:** Correlation matrix showing the association between migraine severity scores (VAS and MIDAS), disease characteristics and hormones

	MIDAS (Arabic)		VAS scale	
	Rho	*P* value	Rho	*P* value
BMI	-0.277	0.069	-0.153	0.321
Age at onset	-0.148	0.336	-0.060	0.700
Duration of headache (years)	0.079	0.61	-0.034	0.825
Duration of attack (hours)	-0.285	0.061	0.024	0.879
Frequency (days per month)	0.183	0.233	0.057	0.712
Longest headache free period (days)	0.148	0.336	0.218	0.154
Number of days on analgesics per month	0.221	0.15	-0.004	0.977
FSH mIU/ml	0.196	0.202	0.088	0.569
Estradiol pg./ml	-0.406	0.007*	-0.331	0.030*

BMI: body mass index, FSH: follicular stimulating hormone, mIU: milli international units, pg.: picogram, ml: milliliter

*P value < 0.05 is considered significant

The linear correlation between two quantitative variables was assessed by Spearman correlation test

### Correlation between migraine severity scores, patient characteristics and hormones

The most notable and statistically significant finding is a negative correlation between age and both MIDAS (Ρ = -0.321, *P* = 0.034) and VAS scale (Ρ = -0.490, *P* = 0.001). Another notable and statistically significant finding is a negative correlation between Estradiol level and MIDAS (Ρ = -0.406, *P* = 0.007) and VAS scale (Ρ = -0.331, *P* = 0.030). Other variables, such as BMI, age at migraine onset, duration of migraine history, duration of attacks, frequency of attacks, headache-free intervals, analgesic use, and FSH, showed weak or no statistically significant correlations with VAS or MIDAS. Although the duration of attacks showed a borderline inverse correlation with MIDAS (Ρ = -0.285, *P* = 0.061), it did not reach significance. Similarly, the correlation between BMI and MIDAS was not statistically significant (Ρ = -0.277, *P* = 0.069) **(**Table 6**).**

**Table 6:** Comparison of perimenopausal symptoms and hormonal levels between migrainous (Group 1 and 2) and nonmigrainous (Control) participants

				Control (*n* = 22)	Group 1 (*n* = 22) ^c^	Group 2 (*n* = 22) ^c^	*P* value
Age			Median (min, max)	49 (43, 52)	49 (44, 52)	46.5 (44, 51)	0.073
Education	Uneducated		Count (%)	5 (22.7%)	5 (22.7%)	11 (50.0%)	0.195
	Educated		Count (%)	17 (77.3%)	17 (77.3%)	11 (50.0%)	
Occupation	Unemployed		Count (%)	4 (18.2%)	4 (18.2%)	4 (18.2%)	1.000
	Employed		Count (%)	18 (81.8%)	18 (81.8%)	18 (81.8%)	
Menstrual Irregularities			Count (%)	22 (100%)	22 (100%)	22 (100%)	NA
Vaginal Dryness			Count (%)	5 (22.7%)	8 (36.4%)	11 (50.0%)	0.171
Hot Flashes			Count (%)	14 (63.6%)	18 (81.8%)	18 (81.8%)	0.267
Night Sweating			Count (%)	11 (50.0%)	18(81.8%)	16 (72.7%)	0.066
Fatigue			Count (%)	4 (18.2%) ^a^	17 (77.3%) ^b^	16 (72.7%) ^b^	< 0.001^*^
Sleep Changes (insomnia)			Count (%)	6 (27.3%) ^a^	16 (72.7%) ^b^	18 (81.8%) ^b^	< 0.001^*^
Depressed mood			Count (%)	4 (18.2%) ^a^	16 (72.7%) ^b^	16 (72.7%) ^b^	< 0.001^*^
Anxiety			Count (%)	5 (22.7%) ^a^	14 (63.6%) ^b^	12(54.5%) ^b^	0.017^*^
Estradiol (pg./ml)			Median (Min, Max	35.5(13.9, 150) ^a^	19.8 (2.8, 66) ^b^	22.9 (2.9, 75) ^b^	0.018^*^
FSH (mIU/ml)			Median (Min, Max)	33.8(2.9, 88.7)	33.8(2.9, 88.7)	19.8(2.5, 90.5)	0.921
			Total				
Estradiol < 50pg/ml		Count (%)	56 (84.9%)	15 (68.2%)	21 (95.4%)	20 (90.9%)	0.016*
FSH > 30mIU/ml		Count (%)	37 (56.1%)	11 (50%)	14 (63.6%)	12 (54.5%)	0.315

Comparing between more than two groups, Kruskal Wallis test was used for quantitative variables while Chi-squared test was used to compare categorical variables

P value < 0.05 is considered significant

*P value is corrected by Bonferroni correction formula for multiple testing and P value ≤ 0.002 is considered significant

Different superscript letters (a, b, c) mean statistically significant differences among groups (*P* < 0.05), while same letters mean no significant difference

^c^ Group 1 includes perimenopausal patients who were proved to have migraine headache with an onset during their perimenopausal period while Group 2 includes perimenopausal patients who were proved to have migraine headache with an onset during their child-bearing age

## Discussion

In this study, the three groups, including migrainous participants and control groups, were similar in their basic demographic characteristics. These similarities suggest that demographic factors likely did not confound the analysis of symptoms or hormonal profiles.

In this study, the perimenopausal period was associated with a high frequency of attacks of migraine and a long duration of headache attacks. This result was agreed upon by Martin and colleagues [17].

In our study, unilateral location with alternating side for headache was the most common in both groups, orbito-fronto-temporal location was the most common site, and throbbing pain was the most common pain nature. Additionally, the most common associated symptoms in order of frequency include photophobia, phonophobia, nausea and/or vomiting, visual symptoms, osmophobia and autonomic manifestations. Furthermore, more than half of migraineurs reported allodynia. Notably, postdrome symptoms (e.g., fatigue, mood changes following migraine resolution) were significantly more common in Group 2 than in Group 1. This could indicate a more pronounced after-effect of migraine episodes in those with long-lasting migraines. However, premonitory symptoms (e.g., yawning, food craving, mood changes, fatigue, thirst) were less common in both groups. These clinical migraine features are similar to literature [16–23].

It was found in this study that about two-thirds of patients have severe migraine disability (MIDAS > 20) and about one-third have moderate migraine disability (MIDAS 11–20). Regarding the assessment of pain intensity by using the VAS scale in this study, it was found that nearly half of the patients are in the severe VAS category (score of 7–10), while the other half are in the moderate category (score of 4–6) in both groups. Li and colleagues in their study found that the mean MIDAS score of migraine during the postmenopausal period was 28.31 ± 16.50, whereas the mean VAS score was 7.45 ± 2.51. These results are comparable to previous studies in the literature [23, 24].

Makita and colleagues in their study reported that 35.7% of study subjects are in the moderate VAS category and 46.2% are in the severe VAS category [25]. This difference can be justified by the difference in the study design and the larger sample size in the Japanese study (171 women compared to 66 women in our study), which can affect the statistical analysis of the results.

Both migraine groups in this study showed similar patterns of migraine triggers, including stress, exertion, fatigue, hot weather, sleep changes and fasting. However, menstrually related migraine is higher in frequency in Group 2 with a statistically significant difference (*p* = 0.016). The most common relieving factor was the use of analgesics; with a tendency to overuse over the counter medications denoting the lack of awareness about migraine preventive therapy and increase the risk of medication-overuse headache. Dark room, sleep and bed rest are additional relieving factors reported in this study. These findings are supported by the results of the literature [22, 26, 27].

The most notable hormonal finding in this study was the lower Estradiol levels in migraine participants compared to healthy control participants; this difference was statistically significant with a P value < 0.05. Our results also showed a statistically significant (*P* < 0.05) negative correlation between Estradiol level and migraine scores (MIDAS and VAS).

However, FSH levels didn’t show a statistically significant difference between study groups.

These results agree with the already known estrogen withdrawal theory, which is a widely accepted hypothesis that offers an explanation for how migraine can be triggered by female sex hormones. According to this hypothesis, migraine attacks can be induced just before menstruation, during menopausal transition and/ or in the early postmenopausal period [28]. This result is also agreed upon by a previous study, which concluded that postmenopausal estrogen level was negatively correlated with migraine frequency and MIDAS score [23]. Our findings were different from the findings of a cross-sectional study conducted in China. This study mentioned that lower migraine burden was related to the presence of low Estradiol (˂50 pg./mL) and high FSH levels (˃30 mIU/mL), even in the premenopausal and early perimenopausal women. This difference can be explained by different races, sample size and methodology [29].

Regarding the association between FSH level and migraine, there is limited data and data heterogeneity in the literature [23, 29, 30].

It was reported in the literature that migraineur women may suffer from a higher frequency of climacteric vasomotor symptoms than non-migraineurs, which may be related to some share in the pathogenesis of migraine and vasomotor symptoms, including hormonal factors and CGRP [31, 32]. Similarly, in our study, we found a high burden of vasomotor symptoms (hot flashes, night sweating, vaginal dryness, fatigue, sleep disturbances, mood symptoms and anxiety) in migrainous women compared to control healthy participants.

There are a number of limitations in our study. Firstly, the small sample size may limit the generalizability of the results. Secondly, this study is a clinic-based study. Patients who seek clinic care usually have moderate to severe, high-frequency headache or analgesic nonresponsive headache. Whereas those who have mild headaches or headaches that respond to over-the-counter medications usually will not seek help for their headaches. This limitation can cause selection bias. Thirdly, data were collected based on participants’ self-reports (participants were new clinic-attenders) about the severity and frequency of headache rather than the use of a headache diary, which can cause recall bias. Fourthly, the time from the last menstrual period was not recorded individually for each participant, and we acknowledge this as a limitation that can be a source of potential bias. Finally, the samples of hormonal levels were collected regardless of menstrual timing because of the menstrual irregularities. Lastly, this study didn’t include an MRI brain due to limited resources and funds.

### Recommendations

A Large population-based or multicenter cohort study is recommended to explore migraine features during the perimenopausal period and the relationship between sex hormones and migraine. We also recommend precisely recording the last menstrual period (LMP) during data collection in future studies during the perimenopausal period for more accurate characterization of the perimenopausal stage. Actually, a multidisciplinary care is recommended for perimenopausal women with migraine, including a neurologist, a gynecologist and a psychiatrist. To provide a more specialized headache care for patients who reside far from major cities, specialized headache clinics should be made available to the whole population. Another recommendation is to help raise awareness of using migraine preventive therapy, which can reduce the dilemma of medication overuse headache and migraine chronicity during perimenopause.

## Conclusion

Most of the clinical characteristics of perimenopausal migraine are similar to those of migraine in other age groups. Perimenopause was associated with a high frequency of episodic migraine with moderate to severe attacks. There is an inverse relationship between migraine disability and intensity scores and Estradiol level. The frequency of perimenopausal symptoms is higher, while the Estradiol level is lower in migrainous compared to nonmigrainous perimenopausal women.

## Data Availability

The datasets used and analyzed during this study are available from the corresponding author upon formal request.

## Declarations

### AI tools

We acknowledge that the English text of this manuscript was proofread and corrected using an AI-powered tool (Advanced Grammarly tool)

## Abbreviations

CGRP: Calcitonin Gene-Related Peptide
FSH: Follicular Stimulating Hormone
HRT: Hormonal Replacement Therapy
ICHD: International Classification of Headache Disorders
MIDAS: Migraine Disability Assessment Score
NICE: National Institute for Health and Care Excellence
PACAP: Pituitary Adenylate Cyclase-Activating Polypeptide
VAS: Visual Analogue Scale
